# Intracranial pressure responsiveness to positive end-expiratory pressure is influenced by chest wall elastance: a physiological study in patients with aneurysmal subarachnoid hemorrhage

**DOI:** 10.1186/s12883-018-1132-2

**Published:** 2018-08-24

**Authors:** Han Chen, Kai Chen, Jing-Qing Xu, Ying-Rui Zhang, Rong-Guo Yu, Jian-Xin Zhou

**Affiliations:** 1 Department of Critical Care Medicine, Beijing Tiantan Hospital, Capital Medical University, No 6, Tiantan Xili, Dongcheng District, Beijing, China; 2 Surgical Intensive Care Unit, Fujian Provincial Clinical College, Fujian Medical University, Fuzhou, Fujian China

**Keywords:** Respiratory mechanics, Chest wall elastance, Intracranial pressure, Positive end-expiratory pressure, Esophageal pressure

## Abstract

**Background:**

Respiratory system elastance (E_RS_) is an important determinant of the responsiveness of intracranial pressure (ICP) to positive end-expiratory pressure (PEEP). However, lung elastance (E_L_) and chest wall elastance (E_CW_) were not differentiated in previous studies. We tested the hypothesis that patients with high E_CW_ or a high E_CW_/E_RS_ ratio have greater ICP responsiveness to PEEP.

**Methods:**

An esophageal balloon catheter was placed to measure esophageal pressure. PEEP was increased from 5 to 15 cmH_2_O. Airway pressure and esophageal pressure were measured and E_L_, E_CW_ and E_RS_ were calculated at the two PEEP levels. Patients were classified into either an ICP responder group or a non-responder group based on whether the change of ICP after PEEP adjustment was greater than or less than the median of the overall study population.

**Results:**

The magnitude of the increase in esophageal pressure (median [interquartile range]) at end-expiratory occlusion was significantly increased in the responder group compared with that in the non-responder group (4.1 [2.7–4.1] versus 2.7 [0.0–2.7] cmH_2_O, *p* = 0.033) after PEEP adjustment. E_CW_ and the E_CW_/E_RS_ ratio were significantly higher in ICP responders than in non-responders at both low PEEP (*p* = 0.021 and 0.017) and high PEEP (*p* = 0.011 and 0.025) levels. No significant differences in E_RS_ and E_L_ were noted between the two groups at both PEEP levels.

**Conclusions:**

Patients with greater ICP responsiveness to increased PEEP exhibit higher E_CW_ and a higher E_CW_/E_RS_ ratio, suggesting the importance of ECW monitoring.

**Electronic supplementary material:**

The online version of this article (10.1186/s12883-018-1132-2) contains supplementary material, which is available to authorized users.

## Background

Acute lung injury is prevalent in patients with acute brain injury [1–4]. Mechanical ventilation is needed in this population, and positive end-expiratory pressure (PEEP) is often used to improve oxygenation and prevent or recruit alveolar collapse [5–7]. However, there have long been concerns that the use of PEEP in brain-injured patients could reduce cerebral perfusion pressure due to both increased intracranial pressure (ICP) and decreased mean arterial pressure [8, 9]. Previous studies yielded inconsistent effects of PEEP on ICP [10] and diverse individual responsiveness [11–13], suggesting that the mechanism of action might be multifactorial. Several possible determinants for the influence of PEEP on ICP have been proposed, including baseline ICP [14], intracranial compliance [15, 16], respiratory mechanics [16, 17], dead space change and alveolar recruitment by PEEP [18].

Theoretically, PEEP may increase ICP by reducing venous return via elevating intrathoracic pressure [9]. Thus, ICP responsiveness to PEEP might largely depend on pressure transmission from the lung to the pleural cavity, which is determined by the distribution of lung elastance (E_L_) and chest wall elastance (E_CW_) in respiratory system elastance (E_RS_). Although clinical studies have suggested that an increased E_RS_ might attenuate the effect of PEEP on ICP, E_L_ and E_CW_ were not differentiated in these studies [16, 17]. A given increased E_RS_ might mainly contribute to the increase in E_L_ due to pulmonary causes, such as acute respiratory distress syndrome (ARDS), or the increase in E_CW_ due to the chest wall impairment, such as intra-abdominal hypertension or massive pleural effusion [19]. In mechanical ventilated patients, the reported E_CW_ to E_RS_ ratio (E_CW_/E_RS_ ratio) varied from 0.2 to 0.8 [19]. To date, no study has been performed to determine the different effects of E_L_ and E_CW_ on ICP responsiveness to PEEP, which is worthy of investigation.

We hypothesized that the patients with high E_CW_ or a high E_CW_/E_RS_ ratio might exhibit more significant ICP responsiveness to PEEP. In the present study, patients with aneurysmal subarachnoid hemorrhage after clipping surgeries were enrolled, and two different levels of PEEP were applied. Changes in ICP after PEEP adjustment were monitored. Esophageal pressure was measured as a surrogate for pleural pressure, and the distribution of E_L_ and E_CW_ in E_RS_ was determined. The aim was to investigate the possible influencing factors in ICP responsiveness to PEEP, especially for E_CW_ and the E_CW_/E_RS_ ratio.

## Methods

### Ethics and setting

This study was conducted in the Surgical Intensive Care Unit of Fujian Provincial Hospital, Fuzhou, China. The study protocol was approved by the Institutional Review Board of Fujian Provincial Hospital (K2015–023-01) on September 30, 2015, and the study was registered at ClinicalTrials.org (NCT02670733) on January 26, 2016 [20]. Because the study enrolled patients that were in a coma state, written informed consent was obtained from patient’s appropriate substitute decision maker designated to provide consent upon admission to the hospital.

### Patients

All adult patients receiving aneurysm clipping after aneurysmal subarachnoid hemorrhage were consecutively screened daily. The inclusion criteria included 1) Glasgow Coma Scale (GCS) ≤ 8; 2) ventricular ICP monitor previously placed for ICP monitoring and cerebrospinal fluid drainage during the operation; and 3) need for mechanical ventilation with PEEP. The exclusion criteria were 1) age under 18 years; 2) after decompressive craniectomy; 3) ICP > 25 mmHg; 4) hemodynamic instability requiring greater than 10 μg/kg/min dopamine or more than 0.5 μg/kg/min norepinephrine; 5) history of esophageal surgery or chronic obstructive pulmonary disease; 6) evidence of active air leak from the lung or existing chest tube; and 7) expected to survive less than 24 h.

Given the lack of a widely accepted threshold to identify the responsiveness of ICP to increased PEEP levels, we classified each patient into two groups according to the median change of ICP after PEEP adjustment in the overall study population: above the median value was considered the “responder” group and below the median value was considered the “non-responder” group. This was an unblinded study, but the researchers were not aware of the patients’ allocation until all data were collected (the median can only be determined by then).

### ICP monitoring and intracranial ventricular compliance measurements

ICP was measured using a ventricular ICP monitor (Codman, Johnson & Johnson, Raynham, MA, USA). Ventricular drainage was blocked during the procedure as long as tolerated by the patient (i.e. without an ICP > 25 mmHg). To measure the ventricular compliance, 2 mL of cerebrospinal fluid was drained, and immediate changes in ICP values were recorded. Ventricular compliance was calculated as the ratio of the cerebrospinal fluid drainage volume to the decrease in ICP after the drainage (mL/mm Hg) [15, 16].

### Study procedure

During the study, the patient remained in a supine position with the head of the bed elevated to 30°. Esophageal pressure was measured by a SmartCath-G adult esophageal balloon catheter (7,003,300, CareFusion Co., Yorba Linda, CA, USA). An occlusion test was used to confirm the proper balloon position [21, 22]. Esophageal pressure and airway pressure were measured by two KT 100D-2 pressure transducers (KleisTEK di CosimoMicelli, Italy, range: +/− 100 cm H_2_O), and flow was measured with a Fleisch pneumotachograph (Vitalograph Inc., Lenexa, KS, USA). The pressure and flow signals were displayed continuously and saved (ICU-Lab 2.5 Software Package, ICU Lab, KleisTEK Engineering, Bari, Italy) on a laptop for further analysis. Details on esophageal pressure monitoring and data collection are presented in the Additional file 1.

After the establishment of esophageal pressure monitoring, the patient was sedated and paralyzed via the intravenous infusion of 5 mg of midazolam, 0.1 mg of fentanyl, and 50 mg of rocuronium. Mechanical ventilation was set as volume-controlled ventilation with a constant flow, an inspiratory to expiratory ratio of 1:2, and a tidal volume (V_T_) of 6 to 8 mL/kg of predicted body weight. The initial respiratory rate was set to maintain the PaCO_2_ at 35 to 45 mmHg. End-tidal carbon dioxide partial pressure (P_ET_CO_2_) was also measured (CAPNOSTAT®, Maquet, Solna, Sweden). Pulse oxygen saturation was maintained above 92% by adjusting the FiO_2_.

Two PEEP levels were tested. The PEEP level was first set to 5 cm H_2_O. After a 30-min stabilization, PaO_2_, PaCO_2_ and P_ET_CO_2_ were simultaneously measured, and the ratio of the alveolar dead space to the tidal volume (V_Dal*v*_/*V*_T_) was calculated. ICP, mean blood pressure, cerebral perfusion pressure, central venous pressure and ventricular compliance were measured. End-expiratory and end-inspiratory occlusions were performed for 3 s each, and the airway pressure and the esophageal pressure during the last second of occlusion were recorded. Thereafter, the PEEP level was increased to 15 cm H_2_O and maintained for 30 min without changes in other ventilation settings. The same sequence of measurements was repeated, with the exception that the ventricular compliance measurement was not performed because cerebrospinal fluid drainage might influence ICP monitoring.

E_RS_, E_L_ and E_CW_ were calculated using a standard formula [19, 23]. The E_CW_/E_RS_ ratio was documented. Detailed procedures, measurements and parameter derivations are presented in the Additional file 1.

The procedure was emergently terminated if ICP increased above 25 mmHg or cerebral perfusion pressure decreased below 50 mmHg.

### Statistical analysis

The primary endpoint was the difference in E_RS_, E_L_, E_CW_ and the E_CW_/E_RS_ ratio in patients with different ICP responsiveness to PEEP. We did not calculate sample size because the differences in these elastance parameters between the two groups remain unknown. Instead, we chose 30, a widely accepted minimal sample size for a physiological study, as the sample size in the present study.

Categorical variables are presented as numbers and percentages and were analyzed by Fisher’s exact test. Continuous variables are presented as the median and inter-quartile range (IQR) and were compared using the Mann-Whitney U test or Wilcoxon matched-pair signed-rank test as appropriate. We used the Scheirer-Ray-Hare test to compare the parameters between the responder and the non-responder groups at low and high PEEP levels [24]. All tests of significance were at the 5% significance level and were two-sided. Analyses were performed with SPSS statistics software (V.20.0 IBM Corporation, New York, USA).

## Results

From February to November 2016, 30 patients were studied. No emergent termination occurred during the procedures. Table 1 presents the main characteristics of the enrolled patients at the low (5 cm H_2_O) and high (15 cm H_2_O) PEEP levels. After the PEEP level was increased, ICP significantly increased (*p* <  0.001). The median (IQR) change in ICP was 2.5 (1.0–4.0) mm Hg. According to the predefined criterion, each of the 15 patients was allocated to the “responder” group or the “non-responder” group, which had respective ICP changes of 4.0 (3.0–5.0) and 1.0 (1.0–2.0) mm Hg. Baseline characteristics were comparable between the two groups except for responders with an older age and a lower body mass index (Table 2).

**Table 1:** Patients characteristics at low and high positive end-expiratory pressure

	Low PEEP (5 cmH_2_O)	High PEEP (15 cmH_2_O)	*p*
ICP and hemodynamic parameters			
ICP (mmHg)	4.0 (2.0–10.0)	7.0 (5.8–11.0)	< 0.001
MAP (mmHg)	81.0 (75.6–92.1)	76.2 (67.4–90.0)	0.002
CPP (mmHg)	74.7 (69.6–86.8)	68.7 (60.6–82.7)	< 0.001
CVP (mmHg)	8.0 (5.0–10.0)	12.0 (10.0–14.0)	< 0.001
Respiratory mechanics parameters			
V_T_ (mL/kg)	7.7 (7.1–8.3)	7.7 (7.1–8.2)	0.304
∆P_AW_ (cmH_2_O)	6.8 (6.5–9.5)	9.5 (7.8–10.9)	< 0.001
∆P_CW_ (cmH_2_O)	2.7 (1.4–3.1)	2.7 (2.7–4.1)	0.003
∆P_L_ (cmH_2_O)	4.8 (4.1–5.8)	6.8 (4.1–8.2)	0.003
E_RS_ (cmH_2_O/L)	15.3 (12.5–18.6)	20.1 (16.2–23.9)	< 0.001
E_CW_ (cmH_2_O/L)	5.5 (2.9–6.9)	6.2 (5.4–10.2)	0.001
E_L_ (cmH_2_O/L)	9.4 (7.9–12.1)	12.9 (8.5–15.6)	0.004
E_CW_/E_RS_ ratio	0.33 (0.25–0.43)	0.40 (0.29–0.47)	0.247
Resistance (cmH_2_O/L/sec)	13.0 (9.9–14.2)	12.6 (10.1–15.0)	0.894
Blood gas analysis parameters			
pH	7.40 (7.36–7.44)	7.42 (7.38–7.45)	0.951
PaO_2_ (mmHg)	105.5 (78.0–153.5)	115.5 (78.4–163.5)	0.058
PaO_2_/FiO_2_	264 (180–384)	289 (185–388)	0.061
PaCO_2_ (mmHg)	35.3 (32.6–36.7)	35.3 (33.6–38.2)	0.052
P_ET_CO_2_ (mmHg)	29.5 (27.8–31.2)	29.0 (25.8–30.3)	0.003
V_Dalv_/V_T_ (%)	13.2 (8.5–19.9)	18.8 (11.9–27.7)	< 0.001

Data are shown as median (interquartile range)

*ICP* intracranial pressure; *MAP* mean arterial pressure; *CPP* cerebral perfusion pressure; *CVP* central venous pressure; *V*_*T*_ tidal volume; ∆*P*_*AW*_ airway driving pressure; ∆*P*_*CW*_ chest wall driving pressure; ∆*P*_*L*_: transpulmonary driving pressure; *E*_*RS*_ respiratory system elastance; *E*_*CW*_ chest wall elastance; *E*_*L*_ lung elastance; *PETCO*_*2*_ end-tidal carbon dioxide partial pressure; *V*_*Dalv*_*/V*_*T*_: ratio of the alveolar dead space to the tidal volume

**Table 2:** Patients characteristics at study entry

	All patients (*n* = 30)	Responders (*n* = 15)	Non-responders (*n* = 15)	*p* ^*^
Age (years)	55 (37–66)	66 (42–73)	42 (37–57)	0.045
Male	21 (70)	10 (67)	11 (73)	0.500
BMI (kg/m^2^)	22.8 (22.0–24.2)	22.0 (21.2–23.6)	23.6 (22.0–24.2)	0.039
GCS	5.5 (4.0–6.3)	5.0 (4.0–7.0)	6.0 (5.0–6.0)	0.513
SAPS II	49.5 (46.3–54.6)	49.7 (44.0–57.0)	49.0 (47.0–54.0)	0.400
MV duration (days)^a^	1.0 (1.0–2.0)	1.0 (1.0–2.0)	2.0 (1.0–2.0)	0.345
ARDS diagnosis	6 (20)	4 (27)	2 (13)	0.361
PaO_2_	105.5 (78.0–153.5)	95.0 (76.7–130.0)	129.0 (78.4–168.0)	0.242
PaO_2_/FiO_2_	264 (180–384)	274 (156–325)	323 (196–420)	0.144
PaCO_2_ (mmHg)	35.3 (32.6–36.7)	35.4 (32.7–38.1)	35.1 (30.9–36.1)	0.307

Data are presented as median (interquartile range) for continuous variables and counts (percentages) for categorical variables

*ARDS* acute respiratory distress syndrome, *BMI* body mass index, GCS Glasgow Coma Scale, *MV* mechanical ventilation, *SAPS II* Simplified Acute Physiology Score II

^*^p value in comparison between the responder and non-responder group

^a^MV duration before enrolment

Detailed data of respiratory mechanics, ICP and hemodynamic parameters and blood gas analysis are presented in the Additional file 2 (Table E1 to E6).

**Fig. 1:**
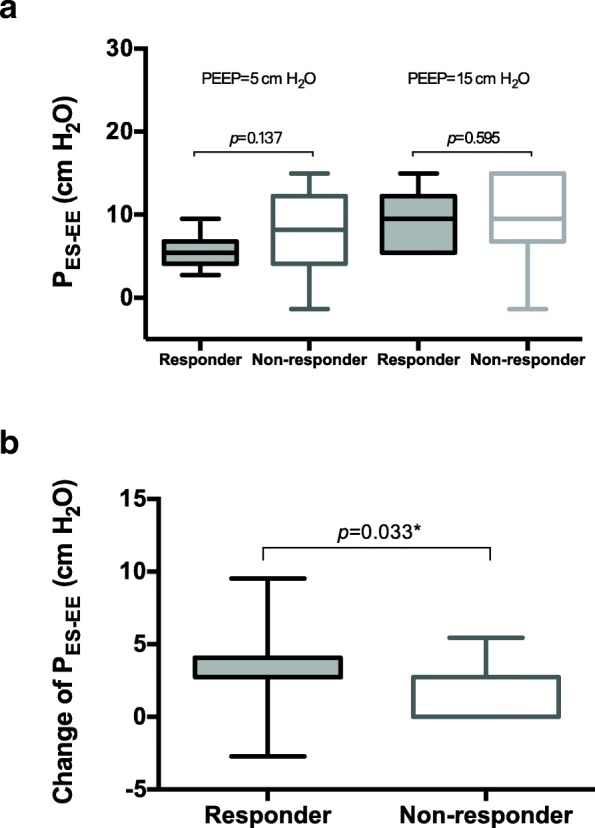
End-expiratory esophageal pressure (P_ES-EE_) measurements at different positive end-expiratory pressure (PEEP) levels. **a** The absolute values of P_ES-EE_ in the responder and non-responder groups. No significant difference was observed between the two groups. **b** The change of P_ES-EE_ during the adjustment of PEEP. The change of P_ES-EE_ in the responders was significantly increased compared with that in non-responders, suggesting that more pressure was transmitted to the pleural cavity

**Fig. 2:**
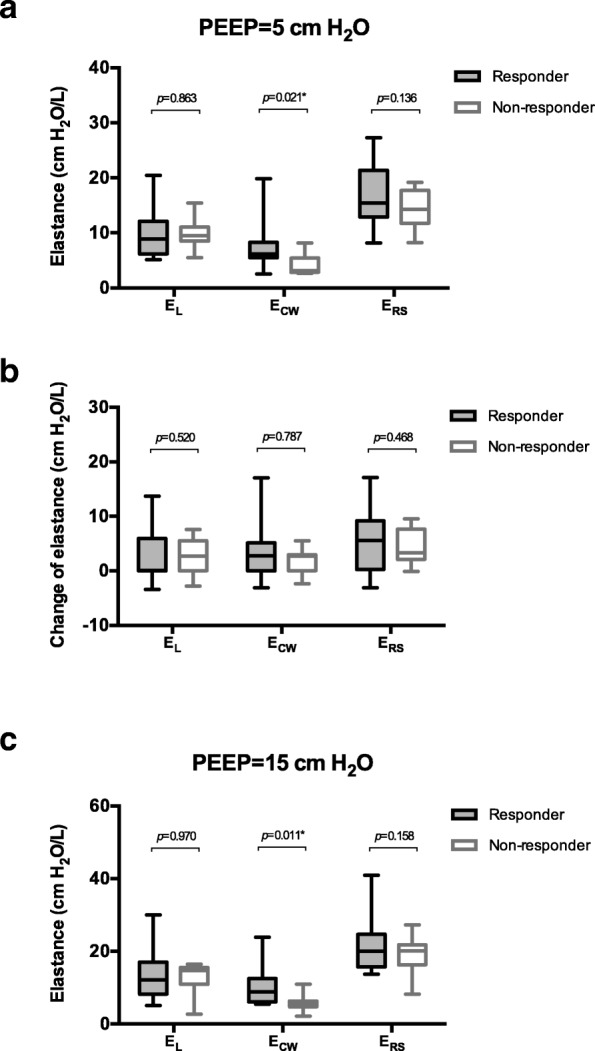
Elastance of the lung, the chest wall and the respiratory system at different positive end-expiratory pressure (PEEP) levels. **a** Elastance measured at a PEEP level of 5 cm H_2_O. Chest wall elastance (E_CW_) was significantly increased in responders compare with that in non-responders. There were no significant differences in respiratory system elastance (E_RS_) and lung elastance (E_L_) between the two groups. **b** The change of E_L_, E_CW_ and E_RS_ during the adjustment of PEEP. No significant difference was observed between the two groups. **c** E_L_, E_CW_ and E_RS_ measured at a PEEP level of 15 cm H_2_O. E_CW_ remained significantly increased in responders compared with that in non-responders. There were no significant differences in E_RS_ and E_L_ between the two groups

Esophageal pressure at end-expiratory occlusion increased significantly after PEEP adjustment (5.4 [4.1–8.2] versus 9.5 [6.8–12.2] cm H_2_O, *p* <  0.001), and the magnitude of the increase was significantly higher in the responder group than in the non-responder group (4.1 [2.7–4.1] versus 2.7 [0.0–2.7] cm H_2_O, *p* = 0.033, Fig. 1). At the low PEEP level, there were no significant differences in E_RS_ (*p* = 0.136) and E_L_ (*p* = 0.863) between two groups, but E_CW_ and the E_CW_/E_RS_ ratio were significantly higher in responders than in non-responders (*p* = 0.021 and 0.017) (Fig. 2a). As the PEEP level increased, no significant difference existed in the magnitude of change in E_RS_, E_L_, and E_CW_ between the two groups (*p* = 0.468 to 0.787, Fig. 2b). Thus, significant differences in E_CW_ and the E_CW_/E_RS_ ratio between the two groups remained at the high PEEP level (*p* = 0.011 and 0.025, Fig. 2c).

**Fig. 3:**
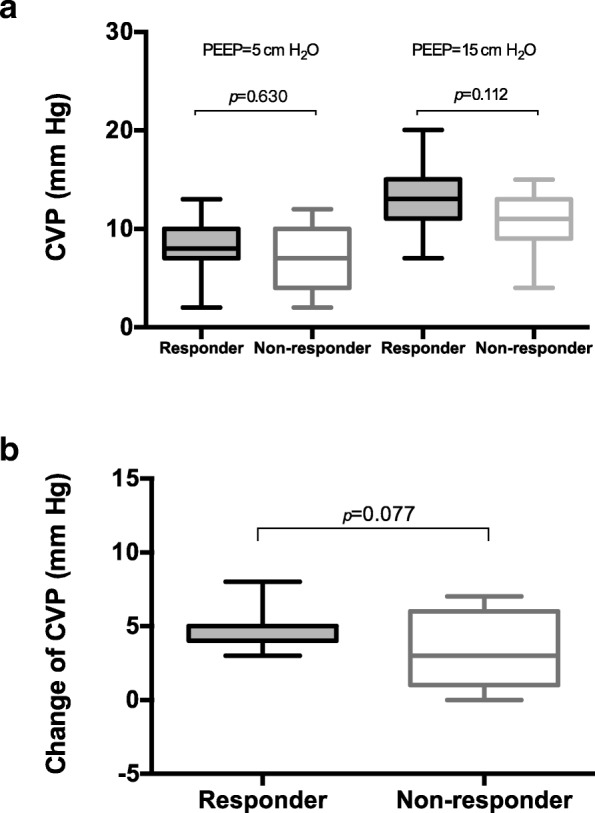
Central venous pressure (CVP) at different positive end-expiratory pressure (PEEP) levels. **a** The absolute values of CVP in the responder and non-responder groups. **b** The change of CVP during the adjustment of PEEP

No significant difference was found in baseline ICP (3.0 [2.0–6.0] versus 4.0 [4.0–10.0] mm Hg, *p* = 0.163) and intracranial ventricular compliance (1.0 [0.7–1.0] versus 1.0 [0.7–1.0] mL/mm Hg, *p* = 0.723) between the ICP responder and non-responder groups. Although central venous pressure increased significantly after the PEEP level was increased in all enrolled patients (Table 1, *p* <  0.001), no significant difference was observed in the change of central venous pressure between ICP responders and non-responders (5.0 [4.0–5.0] versus 3.0 [1.0–6.0] mm Hg, *p* = 0.077, Fig. 3). Similarly, significant decreases in mean blood pressure were observed after PEEP increase in all enrolled patients (81.0 [75.6, 92.1] versus 76.2 [67.4, 90.0], *p* = 0.002), but no difference in the change of mean blood pressure between groups (Table E3 and E4). Cerebral perfusion also decreased after PEEP was increased, the responder had a significantly greater decrease in cerebral perfusion pressure than the non-responder (− 13.0 [− 18.3, − 6.7] versus − 6.7 [− 11.3, 1.0], *p* = 0.011; Table E3 and E4).

There was no significant difference in PaO_2_, PaO_2_/FiO_2_ and PaCO_2_ after PEEP adjustment (*p* = 0.052 to 0.061, Table 1). P_ET_CO_2_ significantly decreased (29.5 [27.8–31.2] versus 29.0 [25.8–30.3] mm Hg, *p* = 0.003); thus, V_Dal*v*_/*V*_T_ significantly increased (13.2 [8.5–19.9] versus 18.8 [11.9–27.7] %, *p* <  0.001). No significant differences were observed in P_ET_CO_2_ and V_Dalv_/V_T_ between ICP responders and non-responders at either the low or high PEEP levels.

## Discussion

The main finding of our study was that patients with a greater ICP responsiveness to increased PEEP exhibit a higher E_CW_ and E_CW_/E_RS_ ratio. This finding was consistent with our hypothesis.

Regarding the influence of PEEP on ICP, clinical studies have reported conflicting results, with ICP increasing [14–18], not markedly changing [25–27] or even decreasing [28] after the application of PEEP. In patients with increased ICP, the effect of PEEP on ICP becomes evident whenever the elevation of the intrathoracic pressure induced by applied PEEP exceeds the ICP [14]. This mechanism was referred to as the Starling resistor model, which represented the role of damaged venous outflow in the response of ICP to PEEP [29]. In a study conducted by McGuire et al., 15 mmHg was used as the upper limit of the normal baseline value of ICP at zero PEEP [14]. ICP increased at PEEP levels of 10 and 15 cm H_2_O in patients with normal baseline ICP but did not significantly change at PEEP levels of 5 to 15 cm H_2_O in patients with elevated baseline ICP. In our group of patients, all ICP values were less than 15 mmHg at 5 cm H_2_O PEEP (4.0 [2.0–10.0] mm Hg) and increased after the PEEP level was adjusted to 15 cm H_2_O (7.0 [5.8–11.0] mm Hg). The difference in ICP at the two PEEP levels (2.5 [1.0–4.0] mm Hg) in our patients was comparable to those previously reported [13, 14].

The PEEP-induced increase of pleural pressure may reduce cerebral venous drainage and eventually increase ICP [9]. The pleural pressure serves as an intermediate link from the lung to the cranium. In the present study, we measured esophageal pressure as a surrogate of pleural pressure and found that esophageal pressure increased more significantly in ICP responders than in non-responders (Fig. 1). This finding suggested a role of pressure transmitted to the pleural cavity in ICP responsiveness. In addition, central venous pressure increased significantly at the high PEEP level (Table 1), indicating the impairment of venous return by PEEP, which eventually resulted in reduced mean blood pressure and cerebral perfusion pressure. ICP responders had a higher tendency to have elevated central venous pressure (Fig. 3); however, statistical significance was not achieved, potentially due to the limited sample size.

The elastic properties of the respiratory system may be a key factor in determining the effect of PEEP on pleural pressure and subsequently ICP [9]. Pressure transmission from the lung to the pleural cavity depends on the distribution of E_L_ and E_CW_ in E_RS_. The higher the proportion of E_CW_ is, the greater the impact of PEEP on pleural pressure [19]. Therefore, E_CW_ and the E_CW_/E_RS_ ratio may be a more important determinant of ICP responsiveness to PEEP. In previous clinical studies, only E_RS_ was used to explore the influence of respiratory mechanics in the PEEP and ICP relationship [16, 17]. After distinguishing E_L_ and E_CW_ from E_RS_, we found that the effect of PEEP on ICP was more profound in patients with higher E_CW_ and a higher E_CW_/E_RS_ ratio (Fig. 2). Although patients with brain injury exhibited higher E_CW_ (without detailed values) in previous studies [30], E_CW_ and the E_CW_/E_RS_ ratio measured in our group of patients were relatively “normal” (Table 1). Our results were comparable to those reported in patients under general anesthesia [31] but higher than those reported in ARDS patients [32]. However, even a slightly higher E_CW_ and E_CW_/E_RS_ ratio might contribute to ICP responsiveness (Fig. 2). Our data suggested the importance of separate E_L_ and E_CW_ monitoring during the clinical application of high PEEP in brain-injured patients.

There are two consequences after the application of PEEP: recruitment of collapsed alveoli and/or overdistention of normal alveoli. In patients with severe brain injury and acute lung injury or ARDS, Mascia et al. defined two groups based on recruitment volume: ≥ 110 mL as recruiters and < 110 mL as non-recruiters [18]. As the PEEP level increased, E_RS_ and PaCO_2_ significantly increased in non-recruiters, indicating hyperinflation. On the contrary, E_RS_ significantly decreased, and PaCO_2_ remained unchanged in recruiters. ICP increased only in non-recruiters. In their patient population, the PaO_2_/FiO_2_ ratio was 95–141 mmHg, and E_RS_ was 24–25 cm H_2_O/L at 5 cm H_2_O PEEP [18]. In our group of patients, only six ARDS cases (20%) were enrolled, and the overall severity of lung injury was mild, as represented by the median PaO_2_/FiO_2_ ratio of 264 and an E_RS_ of 15.3 cm H_2_O/L at the low PEEP level. Alveolar collapse might also be minor; therefore, increases in the PEEP level produced increases in E_RS_ (15.3 to 20.1 cm H_2_O/L) and V_Dal*v*_/*V*_T_ (13.2 to 18.8%). Despite lacking statistical significance, PaCO_2_ and V_Dalv_/V_T_ were high in ICP responders. Therefore, PEEP-induced hyperinflation might also contribute to ICP responsiveness in our patients.

The effect of PEEP on ICP might be affected by ventricular compliance [15, 16]. Apuzzo et al. reported that a significant increase in ICP was only observed in patients who manifested increased cerebral elastance when PEEP was applied [15]. Similarly, Burchiel et al. reported that PEEP increased ICP in patients with decreased intracranial compliance and that decreased lung compliance may buffer this effect [16]. In the present study, given that patients with normal ICP were enrolled and that cerebrospinal fluid was continuously drained before the start of the study, the ventricular compliance was “normal” in all patients. Additionally, for ethical reasons, we cannot measure the ventricular compliance by injecting normal saline into the ventricle instead of draining the cerebrospinal fluid. Because intracranial hypertension and ventricular compliance impairment are prevalent in patients with severe brain injury, further animal experiments should be performed to validate the role of ventricular compliance in the ICP and PEEP relationship.

Increase in PEEP decreases systematic venous return and potentially lowers the cardiac output, this will lower blood pressure and cerebral perfusion pressure. If the cerebral perfusion pressure break through the lower limit of cerebral autoregulation, cerebral perfusion will not be maintained and thus decrease cerebral blood flow, cerebral blood volume and ICP. In this case, the decrease of cerebral perfusion (and thereby decrease in ICP) will somehow contaminate the effect of PEEP on the venous side. In our study, blood pressure and cerebral perfusion pressure were maintained in a normal range, even in a high PEEP level. Therefore, although without cerebral blood flow being measured, we assumed that the cerebral perfusion was comparable between the two groups.

The responders were significant older in this study. It has been reported that the elders have higher E_CW_ due to structural changes to the thoracic cage [33, 34], which is the result of age-related osteoporosis and calcification of the rib cage that reduce the ability of the thoracic cage to expand. Our data also showed higher E_CW_ in the elders, probably due to the same reason. The responders also had significantly lower BMI. Although obesity people have higher E_RS,_ it can be the result of either increased E_L_ or increased E_CW_, or even both [35]. This makes the E_CW_ unpredictable simply base on BMI. Moreover, our patients had relatively normal BMI, the small difference of BMI between the two group (although statistically significant) is unlikely the determinant of the difference of E_CW_.

The main strength of the present study was that the contribution of the chest wall and the lung in ICP responsiveness to PEEP was distinguished. The adverse effect of PEEP on ICP may be amplified in patients with higher E_CW_ and a higher E_CW_/E_RS_ ratio. Our data suggest the potential necessity of respiratory mechanics monitoring when applying PEEP in brain-injured patients, especially in patients with a risk of chest wall impairment.

There were several limitations of the study. First, we used the median of the increased value rather than other physiological parameters to define the ICP responders and non-responders. Given that no widely accepted standard is available to discriminate whether the patient’s ICP is responsive to increased PEEP, the division of patients into two groups is reasonable and enables us to compare potential determinants between the two groups. Second, we only included postoperative subarachnoid hemorrhage patients with relatively normal ICP and respiratory mechanics. Therefore, our results might not be directly applied to other populations. Third, although esophageal pressure has been employed as a surrogate of pleural pressure, the use of the absolute value of esophageal pressure remains questionable [19]. However, in the calculation of E_CW_, we used the change in esophageal pressure between end-inspiration and end-expiration occlusion, which has been shown to reliably reflect the change in pleural pressure [23, 36, 37].

## Conclusions

Patients with greater ICP responsiveness to increased PEEP had higher E_CW_ and a higher E_CW_/E_RS_ ratio. Potential factors contributing to the increase in E_CW_ and the E_CW_/E_RS_ ratio should be assessed and eliminated if possible to avoid the adverse effects of high PEEP levels on the brain.

## Additional files

Additional file 1: Detailed methods of the study procedures, measurements and parameter derivations. (PDF 186 kb)

Additional file 2: Detailed results of respiratory mechanics, intracranial pressure, hemodynamics parameters and blood gas. Values of respiratory mechanics (Table E1), intracranial pressure and hemodynamics parameters (Table E3) and blood gas (Table E5) at low and high positive end-expiratory pressure (PEEP) levels were provided. Changes in respiratory mechanics (Table E2), intracranial pressure and hemodynamics parameters (Table E4) and blood gas (Table E6) from low to high PEEP were also provided. (PDF 177 kb)

## Abbreviations

ARDS: Acute respiratory distress syndrome
CPP: Cerebral perfusion pressure
CVP: Central venous pressure
E_CW_: Chest wall elastance
E_L_: Lung elastance
E_RS_: Respiratory system elastance
ICP: Intracranial pressure
MAP: Mean arterial pressure
P_AW_: Airway pressure
PEEP: Positive end-expiratory pressure
P_ES_: Esophageal pressure
P_ET_CO_2_: End-tidal carbon dioxide partial pressure
V_Dal*v*_/*V*_T_: Alveolar dead space to the tidal volume ratio
V_T_: Tidal volume
