# Supplementary material for: Intracranial pressure responsiveness to positive end-expiratory pressure is influenced by chest wall elastance: a physiological study in patients with aneurysmal subarachnoid hemorrhage
Source: BMC Neurol. 2018 Aug 24;18:124. doi: 10.1186/s12883-018-1132-2 (PMC6108121; doi:10.1186/s12883-018-1132-2)
Supplement: Supplementary file 1 — Detailed methods of the study procedures, measurements and parameter derivations. (PDF 186 kb) [file 12883_2018_1132_MOESM1_ESM.pdf]

Intracranial pressure responsiveness to positive end-expiratory pressure is influenced by chest wall elastance: a prospective physiological study in patients with aneurysmal subarachnoid hemorrhage

Han Chen[1, 2]; Kai Chen[2]; Jing-Qing Xu[2]; Ying-Rui Zhang[2]; Rong-Guo Yu[2]; Jian-Xin Zhou[1*]

[1]: Department of Critical Care Medicine, Beijing Tiantan Hospital, Capital Medical University, Beijing, China

[2]: Surgical Intensive Care Unit, Fujian Provincial Clinical College, Fujian Medical University, Fuzhou, Fujian, China

Additional File 1

Procedure of the placement of esophageal balloon catheter

We used the SmartCath-G adult nasogastric tube with an esophageal balloon (7003300, CareFusion Co., Yorba Linda, CA, USA) in this study. Patients were remained in a supine position with the head of the bed elevated to 30° during the study period. After anesthetizing the nose and oropharynx with 10% lidocaine spray, the esophageal balloon catheter was inserted through the nostril to a depth of 60 cm. The intra-gastric position of the distal part of the catheter was confirmed by aspiration of gastric juice and auscultation of air insufflations into the stomach. After confirmation of the catheter position, the balloon was inflated with 1.5 mL of air, and the proximal part of the catheter was connected to a pressure transducer. Subsequently, the catheter was slowly withdrawn, and the dynamic occlusion test was performed. An end-expiratory occlusion was performed until three to five spontaneous inspiratory efforts were made against the end-expiratory occlusion. The ratio of the change in esophageal pressure (P_{ES}) to the change in airway pressure ($\Delta P_{ES}/\Delta P_{AW}$) was calculated. The catheter was considered

correctly positioned when the $\Delta P_{ES}/\Delta P_{AW}$ ratio during the occlusion test is in the range of 0.8 to 1.2 [1]. In the absence of spontaneous breathing, the positive pressure occlusion test was performed by applying manual compression on the rib cage during the end-expiratory occlusion [2, 3].

Pressure and flow measurements

P_{ES} and P_{AW} were measured by KT 100D-2 pressure transducers (KleisTEK di CosimoMicelli, Italy, range: +/- 100 cm H_2O). Flow was measured with a Fleisch pneumotachograph (Vitalograph Inc., Lenexa, KS, USA) inserted between the Y-piece of the ventilator circuit and the endotracheal tube. The volume was obtained by electrical integration of the flow signal. The Fleisch pneumotachograph and pressure transducers were connected to an ICU-Lab Pressure Box (ICU Lab, KleisTEK Engineering, Bari, Italy) by 80 cm rigid tube lines. The signals were displayed continuously and saved (ICU-Lab 2.5 Software Package, ICU Lab, KleisTEK Engineering, Bari, Italy) on a laptop for further analysis at a sample rate of 200 Hz. The pressure transducers were calibrated with a water column. The pneumotachograph was calibrated with a 1-L calibration syringe (SN: 554-2266, Hans Rudolph, Inc. Shawnee, KS, USA).

Respiratory mechanics measurements

An end-inspiratory occlusion and an end-expiratory occlusion were performed for 3 s. P_{AW} and P_{ES} during the last second of occlusion were recorded. The mean P_{AW} and P_{ES} during a complete cardiac cycle was measured to avoid the influence of cardiac artifacts (Figure E1).

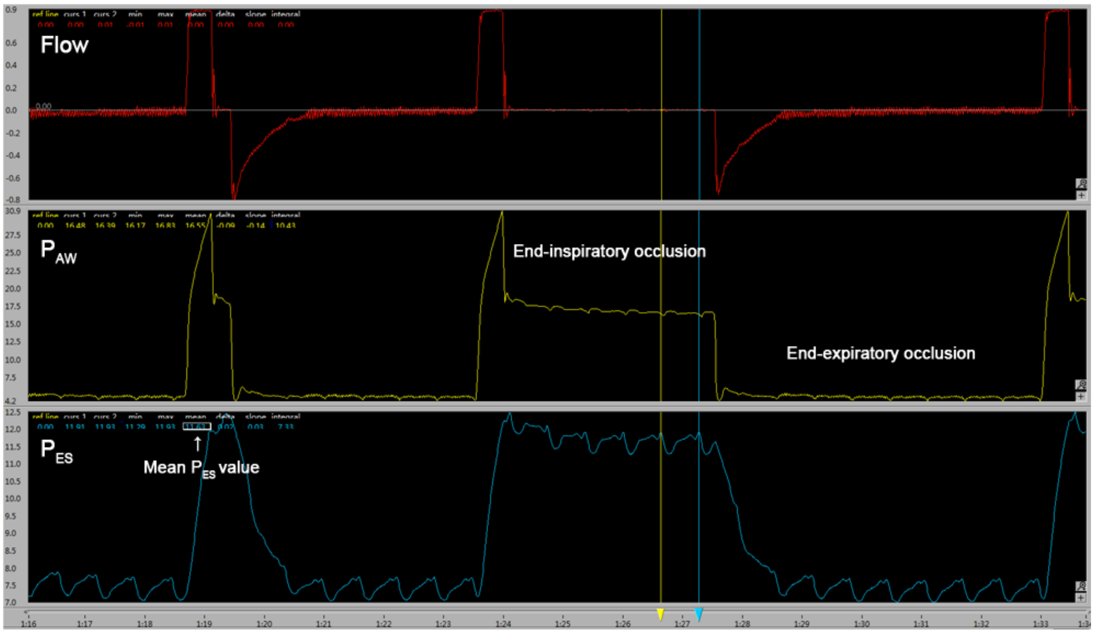

Figure E1 Measurement of airway pressure (P_{AW}) and esophageal pressure (P_{ES}). An approximately 3-s end-inspiratory occlusion and an end-expiratory occlusion were performed. Move the cursors (the yellow and blue vertical lines) to the last and last second peaks, and the the average value between the two cursors were automatically calculated and displayed by the software.

Respiratory system elastance (E_{RS}) can be calculated as:

$$E_{RS} = \frac{P_{PLAT} - PEEP_{TOTAL}}{V_T}$$

Where P_{PLAT} and $PEEP_{TOTAL}$ represent P_{AW} at end-inspiratory and end-expiratory occlusion, respectively. The difference between P_{PLAT} and $PEEP_{TOTAL}$ was called as airway driving pressure (ΔP_{AW}).

E_{RS} can be differentiated into lung elastance (E_L) and chest wall elastance (E_{CW}) [4]:

$$E_{RS} = E_{CW} + E_L$$

Using the P_{ES} as a surrogate for pleural pressure, E_{CW} can be calculated as:

$$E_{CW} = \frac{P_{ES-EI} - P_{ES-EE}}{V_T}$$

Where $P_{ES\text{-}EI}$ and $P_{ES\text{-}EE}$ are respective P_{ES} determined at end-inspiratory and end-expiratory occlusion, and the difference between $P_{ES\text{-}EI}$ and $P_{ES\text{-}EE}$ was called as chest wall driving pressure (ΔP_{ES}).

E_L can be calculated as:

$$E_L = \frac{(P_{PLAT} - P_{ES-EI}) - (PEEP_{TOTAL} - P_{ES-EE})}{V_T}$$

Where (P_{PLAT}-$P_{ES\text{-}EI}$) and ($PEEP_{TOTAL}$-$P_{ES\text{-}EE}$) are the respective transpulmonary pressure at end-inspiratory and end-expiratory occlusion, and the difference of these two parameters represents the transpulmonary driving pressure (ΔP_L).

E_{RS}, E_{CW} and E_L were obtained at the two tested PEEP levels.

References

E1. Baydur A, Behrakis PK, Zin WA et al (1982) A simple method for assessing the validity of the esophageal balloon technique. Am Rev Respir Dis 126:788-791.

E2. Chiumello D, Consonni D, Coppola S et al (2016) The occlusion tests and end-expiratory esophageal pressure: measurements and comparison in controlled and assisted ventilation. Ann Intensive Care 6:13. doi:10.1186/s13613-016-0112-1

E3. Chen H, Xu M, Yang YL et al (2016) Effects of increased positive end-expiratory pressure on intracranial pressure in acute respiratory distress syndrome: a protocol of a prospective physiological study. BMJ Open 6:e012477. doi:10.1136/bmjopen-2016-012477

E4. Gattinoni L, Chiumello D, Carlesso E et al (2004) Bench-to-bedside review: chest wall elastance in acute lung injury/acute respiratory distress syndrome patients. Crit Care 8:350-355. doi:10.1186/cc2854
