# Supplementary material for: Intracranial pressure responsiveness to positive end-expiratory pressure is influenced by chest wall elastance: a physiological study in patients with aneurysmal subarachnoid hemorrhage
Source: BMC Neurol. 2018 Aug 24;18:124. doi: 10.1186/s12883-018-1132-2 (PMC6108121; doi:10.1186/s12883-018-1132-2)
Supplement: Supplementary file 2 — Detailed results of respiratory mechanics, intracranial pressure, hemodynamics parameters and blood gas. Values of respiratory mechanics (Table E1), intracranial pressure and hemodynamics parameters (Table E3) and blood gas (Table E5) at low and high positive end-expiratory pressure (PEEP) levels were provided. Changes in respiratory mechanics (Table E2), intracranial pressure and hemodynamics parameters (Table E4) and blood gas (Table E6) from low to high PEEP were also provided. (PDF 177 kb) [file 12883_2018_1132_MOESM2_ESM.pdf]

Intracranial pressure responsiveness to positive end-expiratory pressure is influenced by chest wall elastance: a prospective physiological study in patients with aneurysmal subarachnoid hemorrhage

Han Chen[1, 2]; Kai Chen[2]; Jing-Qing Xu[2]; Ying-Rui Zhang[2]; Rong-Guo Yu[2]; Jian-Xin Zhou[1*]

[1]: Department of Critical Care Medicine, Beijing Tiantan Hospital, Capital Medical University, Beijing, China

[2]: Surgical Intensive Care Unit, Fujian Provincial Clinical College, Fujian Medical University, Fuzhou, Fujian, China

Additional File 2

Content

1) **Table E1** Respiratory mechanics at low and high positive end-expiratory pressure

2) **Table E2** Changes in respiratory mechanics from low to high positive end-expiratory pressure level (high-low)

3) **Table E3** Intracranial pressure and hemodynamic parameters at low and high positive end-expiratory pressure

4) **Table E4** Changes in intracranial pressure and hemodynamic parameters from low to high positive end-expiratory pressure level (high-low)

5) **Table E5** Blood gas analysis at low and high positive end-expiratory pressure

6) **Table E6** Changes in blood gas analysis from low to high positive end-expiratory pressure level (high-low)

Table E1 Respiratory mechanics at low and high positive end-expiratory pressure

	Low positive end-expiratory pressure (5 cm H_2O)					High positive end-expiratory pressure (15 cm H_2O)				
	All patients	Responders	Non-responders	*p* value[a]		All patients	Responders	Non-responders	*p* value[a]	*p* value[b]
V_{TE} (mL/kg)	7.7 (7.1, 8.3)	7.7 (7.2, 8.3)	7.7 (7.1, 8.3)	0.935		7.7 (7.1, 8.2)	7.7 (7.1, 8.2)	7.7 (7.1, 8.1)	0.905	0.304
ΔP_{AW} (cm H_2O)	6.8 (6.5, 9.5)	6.8 (6.8, 9.5)	8.2 (5.4, 9.5)	0.606		9.5 (7.8, 10.9)	9.5 (6.8, 10.9)	9.5 (8.2, 10.9)	0.496	< 0.001
ΔP_{CW} (cm H_2O)	2.7 (1.4, 3.1)	2.7 (2.7, 4.1)	1.4 (1.4, 2.7)	0.058		2.7 (2.7, 4.1)	4.1 (2.7, 6.8)	2.7 (2.7, 2.7)	0.019	0.003
ΔP_L (cm H_2O)	4.8 (4.1, 5.8)	4.1 (2.7, 5.4)	5.4 (4.1, 6.8)	0.268		6.8 (4.1, 8.2)	5.4 (4.1, 6.8)	6.8 (5.4, 8.2)	0.330	0.003
E_{RS} (cm H_2O/L)	15.3 (12.5, 18.6)	15.5 (12.9, 21.4)	14.2 (11.7, 17.7)	0.136		20.1 (16.2, 23.9)	20.1 (15.7, 24.7)	20.1 (16.3, 21.8)	0.158	< 0.001
E_{CW} (cm H_2O/L)	5.5 (2.9, 6.9)	6.2 (5.5, 8.3)	3.1 (2.8, 5.4)	0.021		6.2 (5.4, 10.2)	8.9 (6.1, 12.5)	5.7 (4.8, 6.3)	0.011	0.001
E_L (cm H_2O/L)	9.4 (7.9, 12.1)	8.9 (6.2, 12.1)	9.5 (8.5, 11.0)	0.863		12.9 (8.5, 15.6)	12.1 (8.2, 17.0)	14.7 (10.9, 15.6)	0.970	0.004
E_{CW} /E_{RS} ratio	0.33 (0.25, 0.43)	0.40 (0.29, 0.50)	0.29 (0.25, 0.33)	0.017		0.40 (0.29, 0.47)	0.40 (0.38, 0.50)	0.29 (0.25, 0.40)	0.025	0.247
Airway resistance (cm H_2O*sec /L)	13.0 (9.9, 14.2)	12.6 (9.3, 13.5)	13.3 (11.8, 14.3)	0.271		12.6 (10.1, 15.0)	12.9 (10.2, 17.9)	12.5 (9.9, 14.2)	0.133	0.894

Data are presented as median (interquartile range).

[a]The *p* value of inter-group comparison. [b]The *p* value of pre-after comparison (low positive end-expiratory pressure to high positive end-expiratory pressure) in the whole study population.

$V_{TE,}$ expiratory tidal volume; $\Delta P_{AW,}$ airway driving pressure; $\Delta P_{CW,}$ chest wall driving pressure; ΔP_{L}, transpulmonary driving pressure; $E_{RS,}$ respiratory system elastance; $E_{CW,}$ chest wall elastance; E_{L} lung elastance.

Table E2 Changes in respiratory mechanics from low to high positive end-expiratory pressure level (high-low)

	All patients	Responders	Non-responders	*p* value
ΔP_{AW} (cm H_2O)	2.7 (1.4, 4.1)	2.7 (0, 4.1)	1.4 (1.4, 4.1)	0.640
ΔP_{CW} (cm H_2O)	1.4 (0, 1.4)	1.4 (0, 2.7)	1.4 (0, 1.4)	0.861
ΔP_L (cm H_2O)	0.7 (0, 2.7)	0 (0, 2.7)	1.4 (0, 2.7)	0.508
E_{RS} (cm H_2O/L)	5.3 (2.1, 8.4)	5.5 (0.2, 9.2)	3.3 (2.1, 7.6)	0.468
E_{CW} (cm H_2O/L)	2.7 (0, 3.1)	2.7 (0, 5.1)	2.7 (0, 3.0)	0.787
E_L (cm H_2O/L)	1.3 (0, 5.6)	0.1 (0, 6.0)	2.7 (0, 5.5)	0.520
E_{CW}/E_{RS} ratio	0.04 (-0.06, 0.13)	0 (-0.04, 0.11)	0.04 (-0.08, 0.15)	0.950
Airway resistance (cm H_2O*sec /L)	0.1 (-1.4, 0.9)	0.2 (-1.9, 3.2)	0.1 (-1.2, 0.6)	0.852

Data are presented as median (interquartile range).

$\Delta P_{AW,}$ airway driving pressure; $\Delta P_{CW,}$ chest wall driving pressure; ΔP_L, transpulmonary driving pressure; $E_{RS,}$ respiratory system elastance; $E_{CW,}$ chest wall elastance; E_L lung elastance.

Table E3 Intracranial pressure and hemodynamic parameters at low and high positive end-expiratory pressure

	Low positive end-expiratory pressure (5 cm H_2O)					High positive end-expiratory pressure (15 cm H_2O)				
	All patients	Responders	Non-responders	*p* value[a]		All patients	Responders	Non-responders	*p* value[a]	*p* value[b]
HR (beats/min)	91.5 (70.3, 98.3)	78 (67.0, 98.0)	92.0 (90.0, 99.0)	0.059		90.0 (75.8, 101.5)	82.0 (68.0, 98.0)	90.0 (80.0, 104.0)	0.598	0.857
MAP (mm Hg)	81.0 (75.6, 92.1)	83.7 (74.3, 101.0)	80.1 (76.3, 84.3)	0.559		76.2 (67.4, 90.0)	82.3 (66.0, 91.0)	75.0 (73.3, 89.7)	0.716	0.002
ICP (mm Hg)	4.0 (2.0, 10.0)	3.0 (2.0, 6.0)	4.0 (4.0, 10.0)	0.163		7.0 (5.8, 11.0)	7.0 (6.0, 9.0)	6.0 (5.0, 11.0)	0.728	< 0.001
CPP (mm Hg)	74.7 (69.6, 86.8)	79.3 (70.3, 92.3)	72.3 (67.7, 80.3)	0.342		68.7 (60.6, 82.7)	67.7 (57.0, 86.0)	69.0 (61.7, 86.0)	0.650	< 0.001
CVP (mm Hg)	8.0 (5.0, 10.0)	8.0 (7.0, 10.0)	7.0 (4.0, 10.0)	0.630		12.0 (10.0, 14.0)	13.0 (11.0, 15.0)	11.0 (9.0, 13.0)	0.112	< 0.001

Data are presented as median (interquartile range).

[a]The *p* value of inter-group comparison. [b]The *p* value of pre-after comparison (low positive end-expiratory pressure to high positive end-expiratory pressure) in the whole study population.

HR, heart rate; MAP, mean arterial pressure; ICP, intracranial pressure; CPP, cerebral perfusion pressure; CVP, central venous pressure

Table E4 Changes in intracranial pressure and hemodynamic parameters from low to high positive end-expiratory pressure level (high-low)

	All patients	Responders	Non-responders	*p* value
HR (beats/min)	1.0 (-2.0, 8.0)	1.0 (-1.0, 4.0)	1.0 (-10.0, 9.0)	0.870
MAP (mm Hg)	-6.8 (-11.4, -1.1)	-9.0 (-13.3, -3.7)	-5.7 (-9.3, 3.0)	0.115
ICP (mm Hg)	2.5 (1.0, 4.0)	4.0 (3.0, 5.0)	1.0 (1.0, 2.0)	<0.001
CPP (mm Hg)	-11.0 (-14.3, -2.6)	-13.0 (-18.3, -6.7)	-6.7 (-11.3, 1.0)	0.011
CVP (mm Hg)	4.0 (3.0, 5.3)	5.0 (4.0,5.0)	3.0 (1.0,6.0)	0.077

Data are presented as median (interquartile range).

HR, heart rate; MAP, mean arterial pressure; ICP, intracranial pressure; CPP, cerebral perfusion pressure; CVP, central venous pressure

Table E5 Blood gas analysis at low and high positive end-expiratory pressure

	Low positive end-expiratory pressure (5 cm H_2O)					High positive end-expiratory pressure (15 cm H_2O)				
	All patients	Responders	Non-responders	*p* value[a]		All patients	Responders	Non-responders	*p* value[a]	*p* value[b]
pH	7.40 (7.36, 7.44)	7.40 (7.35, 7.43)	7.42 (7.37, 7.44)	0.481		7.42 (7.38, 7.45)	7.38 (7.34, 7.44)	7.42 (7.39, 7.45)	0.228	0.951
PaO_2 (mm Hg)	105.5 (78.0, 153.5)	95.0 (76.7, 130.0)	129.0 (78.4, 168.0)	0.242		115.5 (78.4, 163.5)	98.0 (79.1, 171.0)	125.0 (76.4, 153.0)	0.677	0.058
PaO_2/FiO_2	264 (180, 384)	274 (156, 325)	323 (196, 420)	0.144		289 (185, 388)	242 (179, 403)	313 (191, 383)	0.349	0.061
$PaCO_2$ (mm Hg)	35.3 (32.6, 36.7)	35.4 (32.7, 38.1)	35.1 (30.9, 36.1)	0.307		35.3 (33.6, 38.2)	36.4 (35.0, 40.1)	34.0 (31.4, 36.6)	0.105	0.052
$PETCO_2$ (mm Hg)	29.5 (27.8, 31.2)	29.0 (26.0, 31.0)	30.0 (28.0, 32.0)	0.594		29.0 (25.8, 30.3)	29.0 (24.0, 30.0)	29.0 (28.0, 31.0)	0.533	0.003
V_{Dalv}/V_T (%)	13.2 (8.5, 19.9)	15.1 (11.3, 29.4)	9.1 (7.6, 14.8)	0.149		18.8 (11.9, 27.7)	24.4 (15.7, 30.8)	16.0 (8.8, 23.5)	0.074	< 0.001

Data are presented as median (interquartile range).

[a]The *p* value of inter-group comparison. [b]The *p* value of pre-after comparison (low positive end-expiratory pressure to high positive end-expiratory pressure) in the whole study population.

$PETCO_2$, end-tidal carbon dioxide partial pressure; V_{Dalv}/V_T, ratio of the alveolar dead space to the tidal volume

Table E6 Changes in blood gas analysis from low to high positive end-expiratory pressure level (high-low)

	All patients	Responders	Non-responders	*p* value
pH	0.001 (-0.023, 0.017)	-0.005 (-0.032, 0.008)	0.006 (-0.010, 0.019)	0.067
PaO_2 (mm Hg)	6.2 (-4.0, 19.6)	16.0 (-4.0, 22.6)	4.2 (-4.0, 17.0)	0.267
PaO_2/FiO_2 (mm Hg)	15.4 (-10.0, 47.6)	30.3 (-10.0, 55.0)	10.5 (-10.0, 42.5)	0.367
$PaCO_2$ (mm Hg)	0.8 (-0.5, 2.5)	1.2 (-0.1, 3.0)	0.6 (-1.7, 2.0)	0.184
$PETCO_2$ (mm Hg)	-1.0 (-3.0, 0)	-1.0 (-3.0, 0)	-1.0 (-3.0, 0)	0.935
V_{Dalv}/V_T (%)	3.2 (0.8, 9.0)	3.6 (0.1, 11.2)	2.9 (0.9, 6.9)	0.520

Data are presented as median (interquartile range).

$PETCO_2$, end-tidal carbon dioxide partial pressure; V_{Dalv}/V_T, ratio of the alveolar dead space to the tidal volume
